# Supplementary material for: Effects of Dietary Carbohydrate Concentration and Glycemic Index on Blood Glucose Variability and Free Fatty Acids in Individuals with Type 1 Diabetes
Source: Nutrients. 2024 May 2;16(9):1383. doi: 10.3390/nu16091383 (PMC11085728; doi:10.3390/nu16091383)
Supplement: Supplementary file 1 [file nutrients-16-01383-s001.zip › nutrients-2935564-supplementary.pdf]

Supplementary Figures and Captions

Suppl. Figure S1. Macronutrient percentages of the administered sample diets.

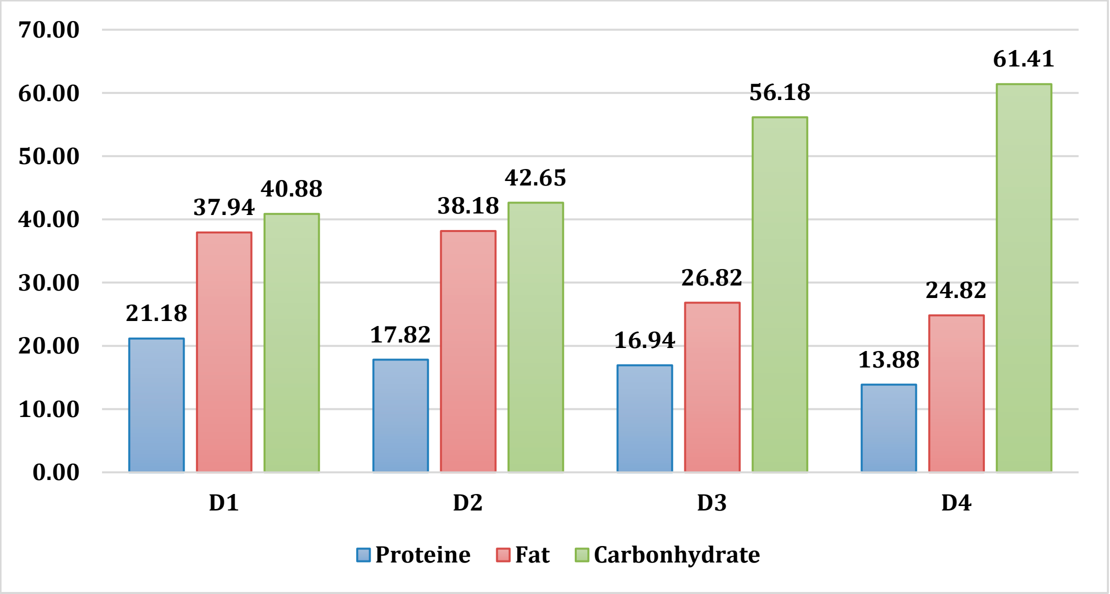

Suppl. Figure S2. Flowchart of the study.

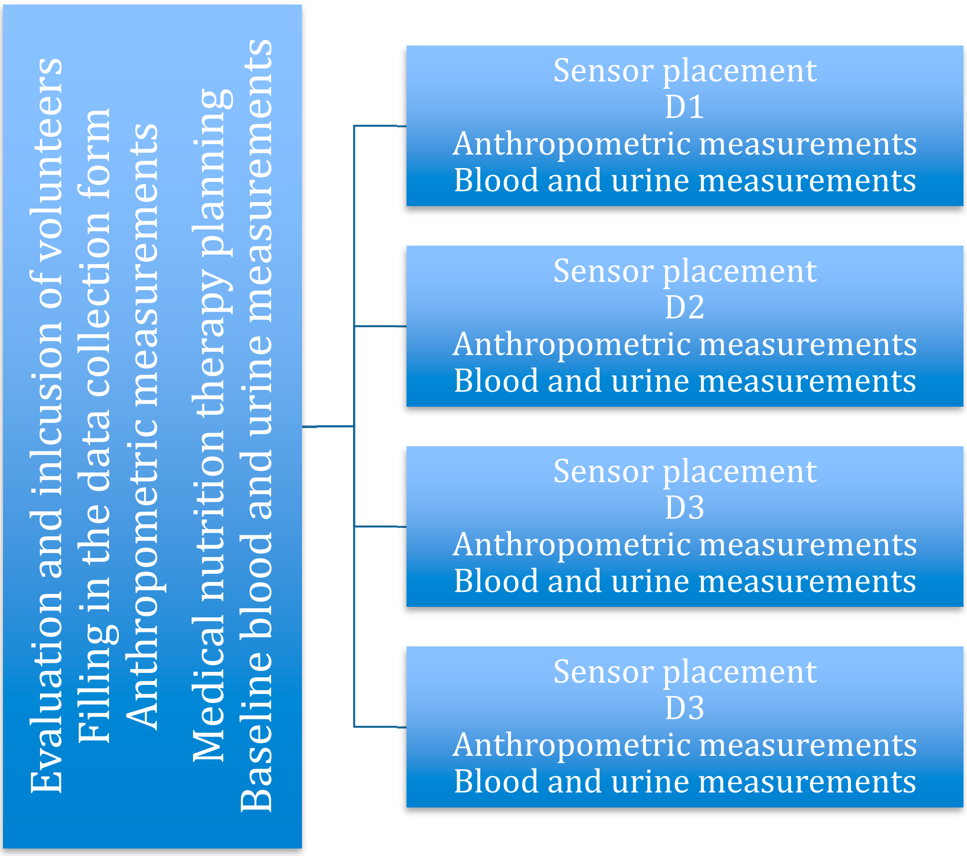

Supplementary Tables and Titles & Footnotes

Suppl. Table S1. Description of the sample diet administered in the study.

Dieet 1	• Low glycemic index diet with 40% carbohydrates tailored to the participant's energy needs
Diet 2	• High glycemic index diet with 40% carbohydrates tailored to the participant's energy needs
Diet 3	• Low glycemic index diet with 60% carbohydrates tailored to the participant's energy needs
Diet 4	• High glycemic index diet with 60% carbohydrates tailored to the participant's energy needs
WASH-OUT	• Medical nutrition therapy with 50% carbohydrate was administered (same as the standardization diet given at the baseline).

Suppl. Table S2. Macronutrient contents of the sample diets administered in the study.

Diets 1 and 2	• The contribution of carbohydrates (avg. 41.2%-42.8%), protein (avg. 18.7%-21.3%) and fat (avg. 37.3%-37.4%) to energy was similar.
Diets 3 and 4	• The contribution of carbohydrates (avg. 57.3%-62.2%), protein (avg. 14.2%-17.0%) and fat (avg. 23.7%-26.5%) to energy was similar.
Diets 1 and 3	• Meals were set with a low glycemic index and similar glycemic index values.
Diets 2 and 4	• Meals were set with a high glycemic index and similar glycemic index values.

Suppl. Table S3. Macronutrient distribution and percentage of the diet groups (%).

	Male		Female		Test statistics, p value	Pairwise comparisons	
	$\bar{X}$	SD	$\bar{X}$	SD			
CHO (g) D1	268.03	44.51	196.21	29.56		D1-D3: p<0.001*	
CHO (g) D2	265.08	35.35	187.09	48.08		D1-D4: p<0.001*	
CHO (g) D3	393	39.9	270.26	31.42		D2-D3: p<0.001*	
CHO (g) D4	396.05	71.24	277.34	27.43			
CHO (%) D1	41.22	2.44	40.5	4.24		3: p<0.001*	D3-D4: p=0.004*
CHO (%) D2	42.78	2.22	42.5	4.34		D1-D4: p<0.001*	
CHO (%) D3	57.33	1	54.88	2.64		D2-D3: p<0.001*	
CHO (%) D4	62.22	1.56	60.5	2.2		D2-D4: p<0.001*	
Protein (g) D1	138.28	19.57	101.09	8.8	Group: F=4.193, p=0.011	D1-D2: p=0.014*	
Protein (g) D2	103.41	25.73	85.59	15.86	Gender: F=4.805, p=0.045	D1-D2: p=0.013*	
Protein (g) D3	101.54	28.25	107.8	27.27	Group*Gender: F=2.417, p=0.08	D1-D4: p=0.029*	
Protein (g) D4	100.46	29.97	89.23	33.51			

Protein (%) D1	21.33	1	21	1.07	Group: F=60.69, p<0.001	2: p<0.001*	D3-D4: p<0.001*
Protein (%) D2	18.78	2.33	16.75	3.01	Gender: F=5.074, p=0.040	D1-D3: p<0.001*	
Protein (%) D3	17	0.5	16.88	1.13	Group*Gender: F=1.227, p=0.30	D1-D4: p<0.001*	
Protein (%) D4	14.22	0.83	13.5	0.93		D2-D4: p<0.001*	
Fat (g) D1	111.16	13.19	85.96	7.86	Group: F=210.8, p<0.001	D1-D3: p<0.001*	
Fat (g) D2	105.61	12.82	77.62	8.97	Gender: F=22.16, p<0.001	D1-D4: p<0.001*	
Fat (g) D3	80.63	9.31	63.58	7.87	Group*Gender: F=7.09, p=0.004	D2-D4: p<0.001*	
Fat (g) D4	69.48	11.33	54.37	7.62		D3-D4: p<0.001*	
Fat (%) D1	37.33	1.8	38.63	3.89	Group: F=166.1, p<0.001<br>Gender: F=14.41, p=0.002<br>Group*Gender: F=0.335, p=0.80	D1-D3: p<0.001*	D3-D4: p=0.004*
Fat (%) D2	37.44	1.94	39	3.46		D1-D4: p<0.001*	
Fat (%) D3	25.56	0.88	28.25	1.58		D2-D3: p<0.001*	
Fat (%) D4	23.78	0.97	26	1.69		D2-D4: p<0.001*	

CHO: carbohydrates, D1: diet 1, D2: diet 2, D3: diet 3, D4: diet 4.

**p<0.001*

Suppl. Table S4. Comparison of the glycemic index and glycemic load averages in dietary interventions for main meals and snacks.

**Measurements**	Mean	**SD**	**Min**	**Max**	**Test statistics, p value**	**Pairwise comparisons**	
Breakfast GL-D1	13.07	4.31	5.46	19.04		D1-D2: p<0.001	
Breakfast GL-D2	32.06	8.1	22.31	48.16	F=80.104,	D1-D3: p=0.005	
Breakfast GL-D3	17.35	4.69	10.2	24.81	p <0.001*	D1-D4: p<0.001	
Breakfast GL-D4	38	9.76	16.95	54.29		D3-D4: p<0.001	
Snack GL-D1	4.43	0.3	3.64	5.29	F=9.029, p=0.002*	D1-D4: p=0.001	
Snack GL-D2	5.33	2.81	0	9.25		D3-D4: p=0.001	
Snack GL-D3	4.59	0.33	44.44	5.29			
Snack GL-D4	7.51	2.46	4.72	11.17			
Lunch GL-D1	20.75	6.81	11.13	38.55		D1-D2: p<0.001	D2-D4: p<0.001
Lunch GL-D2	45.72	15.84	22.21	70.3	F=82.511,	D1-D3: p<0.001	D3-D4: p<0.001
Lunch GL-D3	33.57	10.52	19.11	51.92	p <0.001*	D1-D4: p<0.001	
Lunch GL-D4	75.1	23.02	38.72	118.93		D2-D3: p=0.009	
Mid-afternoon GL-D1	3.84	0.59	3.31	5.29	F=17.157, p <0.001*	D1-D4: p<0.001	
Mid-afternoon GL-D2	5.91	2.93	0.17	12.68		D2-D4: p=0.031	
Mid-afternoon GL-D3	5.31	2.52	3.64	10.58		D3-D4: p=0.002	
Mid-afternoon GL-D4	9.81	3.58	5.68	16.33			
Dinner GL-D1	21.18	6.46	11.13	38.55	F=79.036,	D1-D2: p<0.001	D2-D4: p<0.001

					p <0.001*		
Dinner GL-D2	46.2	15.24	22.23	70.3		D1-D3: p<0.001	D3-D4: p<0.001
Dinner GL-D3	33.77	10.22	19.11	51.92		D1-D4: p<0.001	Dinner GL-D3
Dinner GL-D4	76.46	24.38	38.72	118.93		D2-D3: p=0.007	Dinner GL-D4
Supper GL-D1	5.43	2.1	2.96	9.32	F=9.994, p <0.001*	D1-D4: p=0.002	
Supper GL-D2	7.41	4.27	2.7	17.82		D2-D4: p=0.021	
Supper GL-D3	7.07	1.42	2.96	10.23		D3-D4: p=0.010	
Supper GL-D4	10.53	3.73	5.67	20.93			
Breakfast GI-D1	40.65	2.15	36.24	42.28	F=996.5, p <0.001*	D1-D2: p<0.001	D3-D4: p<0.001
Breakfast GI-D2	75.76	3.05	68.63	79.27		D1-D3: p<0.001	
Breakfast GI-D3	43.86	1.78	39.5	46.39		D1-D4: p<0.001	
Breakfast GI-D4	75.35	2.35	69.3	80.15		D2-D3: p<0.001	
Snack GI-D1	33.41	2.05	31	38.99	F=19.37, p <0.001*	D1-D3: p=0.004	
Snack GI-D2	41.36	14.33	0.14	59.9		D1-D4: p<0.001	
Snack GI-D3	37.06	2.36	36	42		D3-D4: p<0.001	
Snack GI-D4	53.79	5.73	44	63.78			
Lunch GI-D1	32.57	2.73	28.98	38.02	F=2307.0, p <0.001*	D1-D2: p<0.001	Lunch GI-D1
Lunch GI-D2	72.5	1.54	67.74	74.11		D1-D4: p<0.001	Lunch GI-D2
Lunch GI-D3	34.5	2.21	29.89	38.46		D2-D3: p<0.001	Lunch GI-D3
Lunch GI-D4	72	0.83	69.78	73.95		D3-D4: p<0.001	Lunch GI-D4

Mid-afternoon GI-D1	38.3	1.49	36	42	F=5.45, p=0.022*	D1-D4: p=0.006	
Mid-afternoon GI-D2	37.73	12.06	2.9	59.77		D3-D4: p=0.004	
Mid-afternoon GI-D3	38.93	1.79	36.9	42			
Mid-afternoon GI-D4	47.2	8.89	36.83	59.95			
Dinner GI-D1	32.77	2.78	28.98	40.03	F=2183.4, p <0.001*	D1-D2: p<0.001	Dinner GI-D1
Dinner GI-D2	72.5	1.54	67.74	74.11		D1-D4: p<0.001	Dinner GI-D2
Dinner GI-D3	34.47	2.4	29.89	38.46		D2-D3: p<0.001	Dinner GI-D3
Dinner GI-D4	72	0.83	69.78	73.95		D3-D4: p<0.001	Dinner GI-D4
Supper GI-D1	36.43	1.52	35.39	42	F=14.04, p <0.001*	D1-D3: p<0.001	Supper GI-D1
Supper GI-D2	40.82	9.79	30.25	59.77		D1-D4: p=0.001	Supper GI-D2
Supper GI-D3	34.05	0.94	31	35.65		D3-D4: p<0.001	Supper GI-D3
Supper GI-D4	47.47	8.78	31	61.36			Supper GI-D4

D1: diet 1, D2: diet 2, D3: diet 3, D4: diet 4, GI: glycemic index, GL: glycemic load.

*p<0.001

Suppl. Table S5. Energy and carbohydrate contents of the diet groups by gender.

	**Male**		**Female**		**Test statistics, p value**	**Pairwise comparisons**	
	$\bar{X}$	**SS**	$\bar{X}$	**SS**			
Energy (kcal) D1	2664.55	351.9	1996.99	155.39	Group: F=0.663, p=0.58		
Energy (kcal) D2	2502.56	416	1873.91	206.63	Gender: F=17.450, p=0.001		
Energy (kcal) D3	2374.33	482.72	1986.25	160.96	Group*Gender: F=1.953, p=0.14	NA	
Energy (kcal) D4	2303.52	569.08	2147.99	398.26			
CHO (g) D1	268.03	44.51	196.21	29.56		D1-D3: p<0.001*	
CHO (g) D2	265.08	35.35	187.09	48.08		D1-D4: p<0.001*	
CHO (g) D3	393.00	39.90	270.26	31.42		D2-D3: p<0.001*	
CHO (g) D4	396.05	71.24	277.34	27.43			
CHO (%) D1	41.22	2.44	40.5	4.24		D1-D3: p<0.001*	D3-D4: p=0.004*
CHO (%) D2	42.78	2.22	42.5	4.34		D1-D4: p<0.001*	
CHO (%) D3	57.33	1.00	54.88	2.64		D2-D3: p<0.001*	
CHO (%) D4	62.22	1.56	60.50	2.20		D2-D4: p<0.001*	

CHO: carbohydrates, D1: diet 1, D2: diet 2, D3: diet 3, D4: diet 4, NA: not applicable.

*p<0.00
